# Prolonged Gastrointestinal Transit Times and Dysmotility in m.3243A>G Mitochondrial Disease

**DOI:** 10.1111/nmo.70092

**Published:** 2025-06-04

**Authors:** Simone Rask Nielsen, Malene Pontoppidan Stoico, Donghua Liao, Asbjørn Mohr Drewes, Inge Søkilde Pedersen, Anja Lisbeth Frederiksen, Christina Brock

**Affiliations:** ^1^ Department of Clinical Genetics Aalborg University Hospital Aalborg Denmark; ^2^ Department of Clinical Medicine Aalborg University Aalborg Denmark; ^3^ Department of Molecular Diagnostics Aalborg University Hospital Denmark; ^4^ Mech‐Sense, Department of Gastroenterology Aalborg University Hospital Aalborg Denmark; ^5^ Department of Clinical Genetics Odense University Hospital Odense Denmark; ^6^ Department of Clinical Research University of Southern Denmark Odense Denmark

**Keywords:** enteric neuropathy, gastric emptying, gastroenteropathy, gastrointestinal motility, mitochondria, mitochondrial disease, visceral myopathy

## Abstract

**Background:**

Gastrointestinal (GI) symptoms are frequently reported from carriers of the pathogenic mitochondrial DNA variant m.3243A>G, causing decreased mitochondrial adenosine triphosphate (ATP) production. ATP deficiency may adversely affect both autonomic neurogenic and myogenic regulation of GI motility, contributing to the symptoms. This study examined if carriers of m.3243A>G exhibit GI dysmotility, characterized as prolonged segmental transit times and decreased motility indices.

**Methods:**

Twenty‐two Danish carriers of m.3243A>G and 22 healthy, sex‐ and age‐matched controls with body mass index < 26 kg/m^2^ were included. Symptoms were assessed using the validated questionnaires Gastroparesis Cardinal Symptoms Index (GCSI) and Gastrointestinal Symptom Rating Scale (GSRS). GI segmental transit times and motility indices were measured using the ingestible SmartPill, which records pressure, temperature, and pH throughout the GI tract.

**Key Results:**

Median [interquartile range] GI symptoms were higher in carriers of m.3243A>G assessed with GCSI (1.3 [0.4–1.9] vs. 0.2 [0.0–0.4], *p* < 0.001) and GSRS (2.1 [1.4–3.3] vs. 1.1 [1.0–1.2], *p* < 0.001). m.3243A>G carriers further exhibited prolonged median [interquartile range] gastric emptying time (221 min [150–348] vs. 165 min [137–199], *p* = 0.02), colonic transit time (2283 min [1082–5153] vs. 1014 min [840–2451], *p* = 0.03) and decreased mean [confidence interval] colonic motility index (114.7 mmHg*s/min [86.2; 143.3] vs. 168.6 mmHg*s/min [129.9; 207.3], *p* = 0.03) compared to controls. However, these measures were not associated with the perceived GI symptoms.

**Conclusion & Inferences:**

Carriers of m.3243A>G demonstrated gastroenteropathy, evident as prolonged gastric and colonic transit time and decreased colonic motility index. It is plausible that the negative effect of impaired mitochondrial ATP production impacts the regulation of GI motility.

## Graphical abstract

Carriers of m.3243A>G mitochondrial disease have impaired mitochondrial adenosine triphosphate (ATP) production. Our findings indicate that patients exhibit gastrointestinal dysmotility as assessed by the wireless motility capsule SmartPill. This indicates a critical role of mitochondrial ATP in normal gastrointestinal function. Impaired mitochondrial ATP supplies may potentially compromise enteric nerve function and smooth muscle activity.

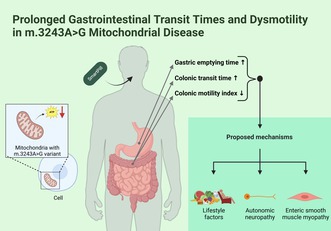

**Summary**

- Carriers of m.3243A>G frequently report gastrointestinal symptoms; the underlying mechanisms driving the symptoms remain, however, poorly understood.
- This study utilized the wireless motility capsule, SmartPill, to evaluate gastrointestinal dysmotility in m.3243A>G carriers, revealing delayed gastric emptying and prolonged colonic transit time, and decreased motility index in the colon.
- Carriers of m.3243A>G mitochondrial disease have impaired mitochondrial function with decreased oxidative phosphorylation and adenosine triphosphate (ATP) synthesis. Our results imply that intact mitochondrial ATP production may be essential for normal gastrointestinal motility.

## 1 Introduction

Vital to energy supplies, the intracellular organelle mitochondria produce cellular energy in the form of adenosine triphosphate (ATP) via oxidative phosphorylation in the respiratory chain. The mitochondria hold their own genome, mitochondrial DNA, and carriers of the pathogenic mitochondrial DNA variant m.3243A>G (MIM 590050) located in the *MT‐TL1* gene have impaired oxidative phosphorylation and reduced mitochondrial ATP production [1]. Impaired mitochondrial ATP production is associated with gastrointestinal (GI) symptoms, as individuals carrying the m.3243A>G variant more frequently report constipation, diarrhea, postprandial fullness, bloating, and abdominal pain compared to healthy subjects [2, 3, 4, 5]. Notably, the mitochondrial genome exists in multiple copies, and pathogenic mitochondrial DNA variants may coexist with wildtype mitochondrial DNA variants in various ratios termed heteroplasmy. For the m.3243A>G variant, increased heteroplasmy level in the blood is generally associated with more severe disease manifestation [6].

GI symptoms negatively impact the m.3243A>G carrier's quality of life [3] and are associated with an increased risk of malnutrition and low body mass index (BMI) [2]. In addition to frequently reported GI symptoms, m.3243A>G carriers also have an increased risk of presenting the rare condition of intestinal pseudo‐obstruction with generalized GI dysmotility, which often has a poor clinical outcome [7, 8, 9]. However, despite the high frequency of GI symptoms reported in carriers of m.3243A>G, the effect of impaired mitochondrial ATP production on intestinal motility and gastric emptying has been insufficiently investigated. Nahora et al. [10] reported delayed gastric emptying in four carriers exhibiting severe GI symptoms. Additionally, Fujii et al. [11] reported reduced gastric motility and delayed gastric emptying in three carriers with gastric symptoms. Furthermore, sporadic case reports and small studies have reported gastroparesis and GI dysmotility in other types of mitochondrial diseases that negatively affect the mitochondrial respiratory chain [12, 13, 14, 15, 16].

Histopathological studies in carriers of m.3243A>G suggest that both neurogenic and myogenic activity within the GI system may be compromised [17, 18, 19]. An intact autonomically regulated and coordinated neurogenic and myogenic function is required to maintain optimal GI function, and the enteric nerves serve to integrate and modulate the intestinal milieu to maintain homeostasis. Interestingly, carriers of the m.3243A>G variant experience symptoms of autonomic neuropathy, including orthostatic intolerance, heat or cold intolerance, incomplete bladder emptying, and pupillary dysfunction in addition to GI disturbances [4]. Moreover, cardiac autonomic neuropathy [20] and autonomic small fiber neuropathy have been reported [21]. Neurons, including those in the enteric nervous system, are crucially dependent on mitochondrial ATP supply for neurotransmitter signaling, maintaining electrochemical gradients across cell membranes, including synaptic functions [22, 23], and for the regulation of axonal Ca^2+^ [24]. Autopsy findings of abnormal large mitochondria in the intestinal ganglionic neurons of m.3243A>G carriers further support the mitochondrial role in neuro‐gastric function [19]. Moreover, recent cell studies utilizing iNeurons derived from human induced pluripotent stem cells, harboring high heteroplasmy levels of m.3243A>G, showed diminished dendritic complexity, a reduced number of synapses, and decreased neural network synchronicity and activity [25]. However, whether an autonomic neuronal impairment is the causal explanation for symptoms remains unclear.

Impaired ATP supplies may also affect smooth muscle cells in the GI tract, even though smooth muscle cells, unlike skeletal muscle, can maintain high tension at a low energy cost [26]. Still, reports of intestinal smooth muscle cells in m.3243A>G carriers showed a high accumulation of structurally abnormal mitochondria [17, 18, 19] and cytochrome oxidase deficiency within the muscular layers of the GI tract [27]. Further, reports of m.3243A>G carriers with high heteroplasmy in GI tissues experienced a high burden of GI symptoms [5, 11, 27, 28, 29]. Such intestinal myopathy may hamper the coordinated muscular forces to move the intestinal content distally, resulting in prolonged transit times and decreased GI motility. To evaluate GI motility, we used the ingestible SmartPill—a wireless motility capsule that records pressure, temperature, and pH throughout the GI tract—enabling segmental investigations based on anatomical landmarks. Thus, we hypothesized that carriers of the m.3243A>G variant and impaired ATP production present GI dysmotility compared to age and sex‐matched healthy controls assessed by segmental transit times, motility index, and contractility parameters as well as investigating the co‐relation between these and subjective GI symptoms.

## 2 Materials and Methods

### 2.1 Study Population

Adult carriers of m.3243A>G recruited from the Dept. of Clinical Genetics, Aalborg University Hospital and from a Danish cohort of known carriers of m.3243A>G [30] were included. Criteria of exclusion were severe dysphagia (inability to ingest the SmartPill), GI obstructions, GI surgery within the past 3 months, Crohn's disease, diverticulitis, implanted or portable electro‐mechanical medical devices (possible mechanical interaction) or known food allergies to the test meal. Any laxatives were paused 3 days before examinations and until the end of the SmartPill investigation. Healthy age‐ and sex‐matched control subjects with a BMI < 26 kg/m^2^ were recruited from the Blood Donation Center, Aalborg University Hospital, and through social media. The study was conducted in accordance with the Declaration of Helsinki and by The North Denmark Region Committee on Health Research Ethics (N‐20210031), and all study participants gave written consent before participation in the study. Data were collected and managed using the REDCap electronic data capture tool (https://redcap.rn.dk; Vanderbilt University, Nashville, TN, USA) hosted at Region Nordjylland.

### 2.2 Assessment of Clinical Factors

Demographic and clinical characteristics, including age, sex, smoking habits, and detailed symptomatic assessment were obtained. The symptomatic assessment included evaluating study participants for diabetes, cardiomyopathy, hypertension, hearing impairment, myopathy, ataxia, epilepsy, stroke‐like episodes, nephropathy, and peripheral neuropathy (patient‐reported glove or stocking sensory loss), which was performed through patient interviews and by assessing the patient journals. Clinical assessment included height and weight. Fasting blood samples were drawn prior to SmartPill ingestion.

### 2.3 Questionnaires Evaluating Gastrointestinal Symptoms

For the assessment of upper and lower GI symptoms, the questionnaires Patient Assessment of Gastrointestinal Disorder Severity and the Gastrointestinal Symptom Rating Scale were recorded by study participants on the day of SmartPill ingestion. Patient Assessment of Gastrointestinal Disorder Severity consists of nine questions scaling from “no symptoms” to “very severe symptoms” using a 2‐week recall period, which covers three main areas (nausea, postprandial fullness, and bloating) in addition to an overall Gastroparesis Cardinal Symptom Index‐score [31]. Gastrointestinal Symptom Rating Scale consists of 15 questions scaling on a seven‐point scale from “No discomfort” to “Very severe discomfort” using a 1‐week recall period, which clusters the questions into five symptoms (reflux, abdominal pain, indigestion, diarrhea, and constipation) in addition to an overall score [32].

### 2.4 SmartPill Investigation

GI segmental transit times and motility indices were obtained with the SmartPill system (SmartPill, Monitoring System; Medtronic, USA) which includes an indigestible single‐use capsule. A “SmartBar” was ingested as a test meal, and a diary was kept of meals, sleeping, and bowel movements until capsule expulsion. The SmartPill measures temperature, pH, and pressure in the intraluminal GI environment as the capsule traverses the GI tract and transmits data to a receiver worn by the study subject [33]. Identification of segmental transit times and motility indexes was performed as suggested by Sarosiek et al. [34] and analyzed using MotiliGI (version 3.0.20; Given Imaging). The motility index consolidates various pressure measurements into a single metric. It was calculated based on the method proposed by Ouyang et al. [35], which involves summing the area under the amplitude curve for contractions exceeding 10 mmHg above baseline. This was determined by multiplying the amplitude of each contraction by its duration, dividing by the time window, and expressing the result in units of mmHg·s/min. Essentially, this approach provides a time‐based summary measure by summing the product of contraction amplitudes and their durations within a given segment. The median pH for each GI segment was calculated. The trans‐pyloric pH rise was defined as the difference in pH from the lowest gastric value to the peak value of the duodenum 30 min before and after gastric emptying. The ileocaecal pH drop was defined as the fall in pH from the peak value in the ileum to the lowest value in the caecum/ascending large bowel 30 min before and after the ileocecal junction. This was performed as proposed by Zarate et al. [36]. The detailed contraction patterns, represented as a number of pressure peaks, were analyzed as suggested by Liao et al. [32]. In summary, contractions were divided into three groups: contractions with a peak pressure > 50 mmHg, contractions with a peak pressure between 50 and 20 mmHg, and contractions with a peak pressure between 10 and 20 mmHg. Pressure amplitudes < 10 mmHg were not included in the analysis. For each contraction pattern group, the ratio between the number of peak pressures within a segment and the transit time of the region, that is, the number of peak pressures per hour, was used to describe the contractility of each segmental region.

### 2.5 Heteroplasmy Level in Blood

Heteroplasmy levels in blood were measured using Droplet Digital Polymerase chain reaction, and data were analyzed using QuantaSoft Analysis Pro 1.0 software (Bio‐Rad, Hercules, California). For further details, see Supporting Information.

### 2.6 Cardiac Vagal Tone

Cardiac vagal tone (CVT) is a parasympathetic measure of the efferent vagal control of the heart, assessed with the linear vagal scale, where 0 represents atropinization and higher values indicate higher parasympathetic adaptable capacities. Following 5 min of rest, cardiac vagal tone was measured for 5 min using a standard 3‐lead electrocardiographic recording (eMotion Faros 180 device [Bittium, Oulu, Finland]). CVT was computed using the algorithm from the ProBioMetrics online app (version 1.0; ProBioMetrics, Kent, United Kingdom) [31]. If the change of two subsequent heartbeats exceeded 15 beats per minute in variation, this was interpreted as a recording artifact due to, for example, coughing or movement and files were cleaned by removing five heartbeat measurements prior to and after the variation artifact. Measurements with more than 20% deleted recording points were excluded from the analysis. As a screening tool for cardiac autonomic neuropathy, the previously described cut off values in type 1 diabetes of < 3.2 linear vagal scales for established and < 5.2 linear vagal scales for borderline cardiovascular autonomic neuropathy were used [37].

### 2.7 Statistical Analysis

Data distribution was assessed using quantile‐quantile plots, and variance homogeneity was assessed using the *F*‐test and, depending on the distribution, presented either as mean ± standard deviation or median and interquartile range. Comparisons of groups of carriers of m.3243A>G and healthy controls for continuous data were performed by a *t*‐test if the observations were normally distributed. Otherwise, the Wilcoxon Mann–Whitney *U*‐test was used for nonparametric data. A chi‐squared test was used to compare the frequency of cardiovascular autonomic neuropathy between m.3243A>G carriers and healthy controls. For the GI motility measures with significant overall differences between groups, the relation within the m.3243A>G carrier group between segmental transit times and GI questionnaire scores, blood heteroplasmy levels, diabetes duration, and hemoglobin A1C (HbA1c) was illustrated using Spearman's or Pearson's correlation coefficient dependent on the distribution of data. In a sub‐analysis, carriers of m.3243A>G were divided into two groups defined by the presence or absence of diabetes, and segmental transit times were compared to their respective age and gender‐matched healthy controls. *p* values < 0.05 was considered statistically significant without adjustment for multiple comparisons as the analyses are explorative. All statistical analyses were performed using STATA statistical software (STATA version 17.0), and graphs were computed using GraphPad Prism version 9.5.

## 3 Results

### 3.1 Participant Characteristics

Twenty‐two carriers of m.3243A>G mitochondrial disease aged 23–63 years (mean 41 years) from 10 different families completed the study. The groups of m.3243A>G carriers and corresponding 22 healthy controls were comparable in age, sex, and BMI. Fifty percent of the carriers of m.3243A>G had diabetes with a mean duration of 12 ± 8 years. Participant characteristics are summarized in Table 1. The main differences included carriers of m.3243A>G having more current smokers, a shorter stature, lower body weight, and elevated plasma lactate, fasting glucose, and triglyceride levels, and carriers of m.3243A>G were more likely to have cardiovascular autonomic neuropathy (*ꭓ* ^2^ [2, *N* = 44] = 12, *p* = 0.002). Twenty‐three percent of the m.3243A>G carriers routinely use laxatives. No adverse events were reported in this study. All segmental transit times were obtained except from one control subject, where a pH drop could not be identified after ingestion of the SmartPill; in contrast, it showed pH and contractile patterns corresponding to the small intestine (direct pyloric shunt) and thus, all gastric measures were excluded from further analysis in this individual. Due to severe data loss (> 20%) in the colonic segment, colonic pH and motility were excluded from one m.3243A>G carrier. In one m.3243A>G carrier, failure of the pH sensor resulted in the exclusion of pH measures from all segments. In two control subjects, the pH sensor was broken in the colonic segment, excluding colonic pH from these subjects. One m.3243A>G carrier had a proton pump inhibitor prior to the investigation and was excluded from analysis for gastric pH and transpyloric pH rise. In two carriers of m.3243A>G, the SmartPill did not exit the body before the receiver ran out of power (> 7 days), and body exit time was defined as the last recorded measurement.

**TABLE 1:** The demographic characteristics of carriers of m.3243A>G and healthy controls.

	Variables	m.3243A>G	Controls	*p*
	Number, *n*	22	22	—
Basic characteristics	Sex (female/male), *n*	15/7	15/7	—
	Age (years)	41 ± 13	41 ± 14	0.94
	Current smokers (yes), *n*	6 (27%)	0 (0%)	
Physical examination	Weight (kg)	61.4 (53.3–73.9)	71.5 (63.7–79.6)	**0.02**
	Height (cm)	168 ± 9	175 ± 11	**0.04**
	Body mass index (kg/m^2^)	22.1 (19.1–25.5)	23.5 (22.6–24.6)	0.21
	Established CAN (yes), *n*	10 (46%)	1 (5%)	**0.002**
	Borderline CAN (yes), *n*	6 (27%)	5 (23%)	
	No CAN (yes), *n*	6 (27%)	16 (73%)	
Biochemistry	Heteroplasmy in blood (%)	24.6 ± 14.3	—	—
	p‐hemoglobin [men 8.3–10.5, women 7.3–9.5 mmol/L]	8.7 ± 1.1	8.4 ± 0.7	0.19
	p‐creatinine [men 60–105, women 45–90 μmol/L]	69 ± 14	68 ± 11	0.73
	p‐lactate [0.5–2.5 mmol/l]	1.5 (1.2–1.9)	0.8 (0.7–1.1)	**< 0.001**
	HbA1c [31–44 mmol/mol]	44 (36–45)	34 (32–34)	**< 0.001**
	HbA1c in diabetics (*n* = 11) (mmol/mol)	45 (44–60)	—	—
	p‐glucose [4.2–7.8 mmol/L]	7.0 (5.8–7.3)	5.3 (5.1–5.5)	**< 0.001**
	p‐cholesterol [< 5.0 mmol/L]	4.5 ± 0.9	4.4 ± 0.9	0.90
	p‐triglycerides [< 2.0 mmol/L]	1.4 (1.0–1.7)	0.7 (0.6–1.0)	**< 0.001**
Medication^a^	Habitual use of laxatives^b^ (yes), *n*	5 (23%)	0 (0%)	—
Symptomatic assessment	Diabetes (yes), *n*	11 (50%)	0 (0%)	—
	Diabetes duration (years)	12 ± 8	—	—
	Cardiomyopathy (yes), *n*	4 (18%)	0 (0%)	—
	Hypertension (yes), *n*	7 (32%)	1 (5%)	—
	Hearing impairment (yes), *n*	14 (64%)	0 (0%)	—
	Myopathy (yes), *n*	14 (64%)	0 (0%)	—
	Ataxia (yes), *n*	2 (9%)	0 (0%)	—
	Epilepsy (yes), *n*	0 (0%)	0 (0%)	—
	Stroke‐like episodes (yes), *n*	0 (0%)	0 (0%)	—
	Nondiabetic nephropathy (yes), *n*	1 (5%)	0 (0%)	—
	Peripheral neuropathy (yes), *n*	8 (36%)	0 (0%)	—

*Note:* Data are presented as mean ± standard deviation, median (interquartile range) or number (%). Normal reference values for biochemistry results are noted in brackets. Significant differences are shown in bold.

Abbreviations: CAN, cardiovascular autonomic neuropathy; HbA1c, hemoglobin A1C; p, plasma.

^a^ For full details on medical history see Table S1.

^b^ Laxatives were paused three days prior to the investigation.

### 3.2 Gastrointestinal Transit Times

**FIGURE 1:**
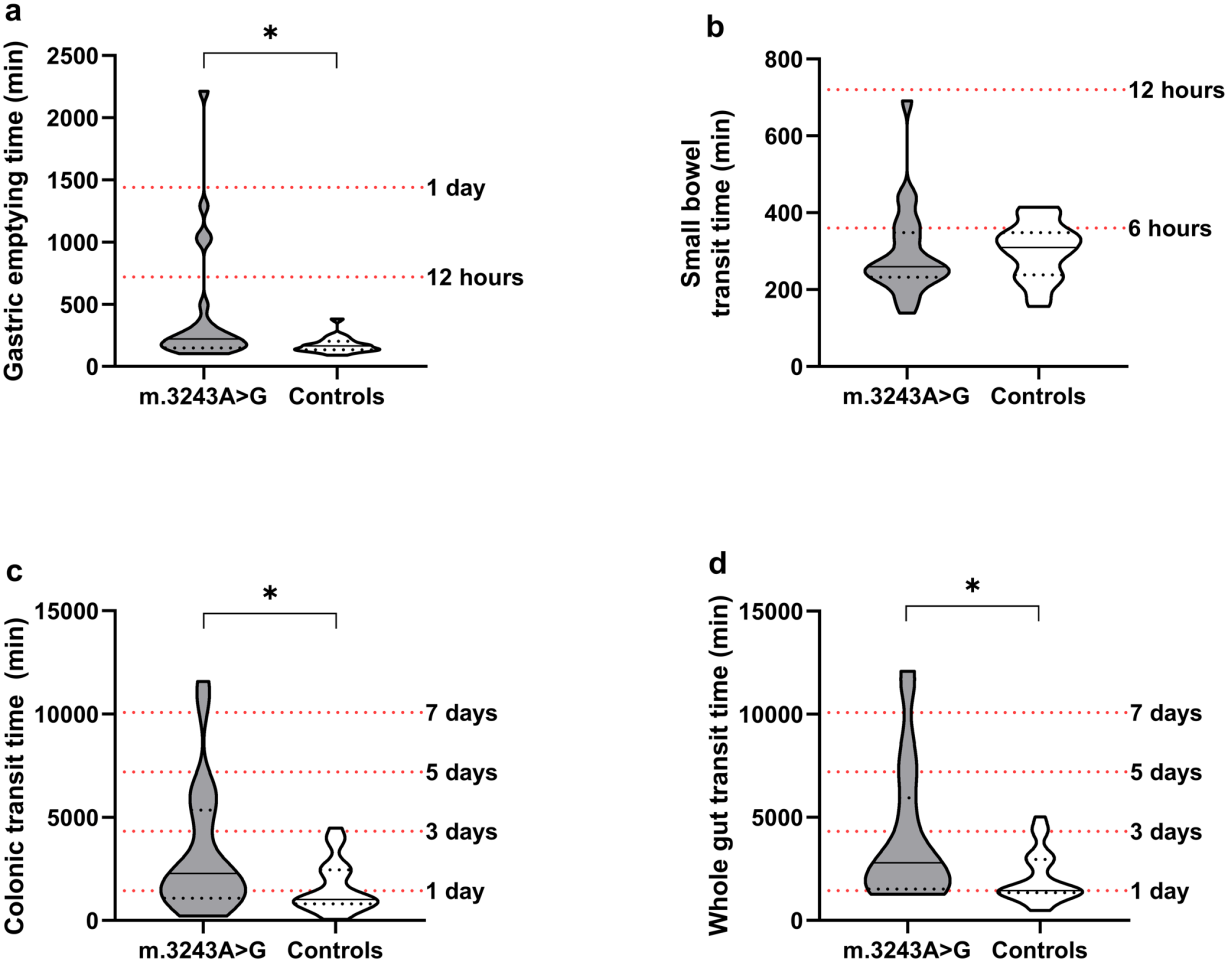
Violin plot with median (straight line) and quartiles (dotted lines) illustrating differences in gastric emptying time (a), small bowel transit time (b), colonic transit time (c) and whole gut transit time (d) between carriers of m.3243A>G and healthy controls. *Illustrate significant differences in transit times.

**FIGURE 2:**
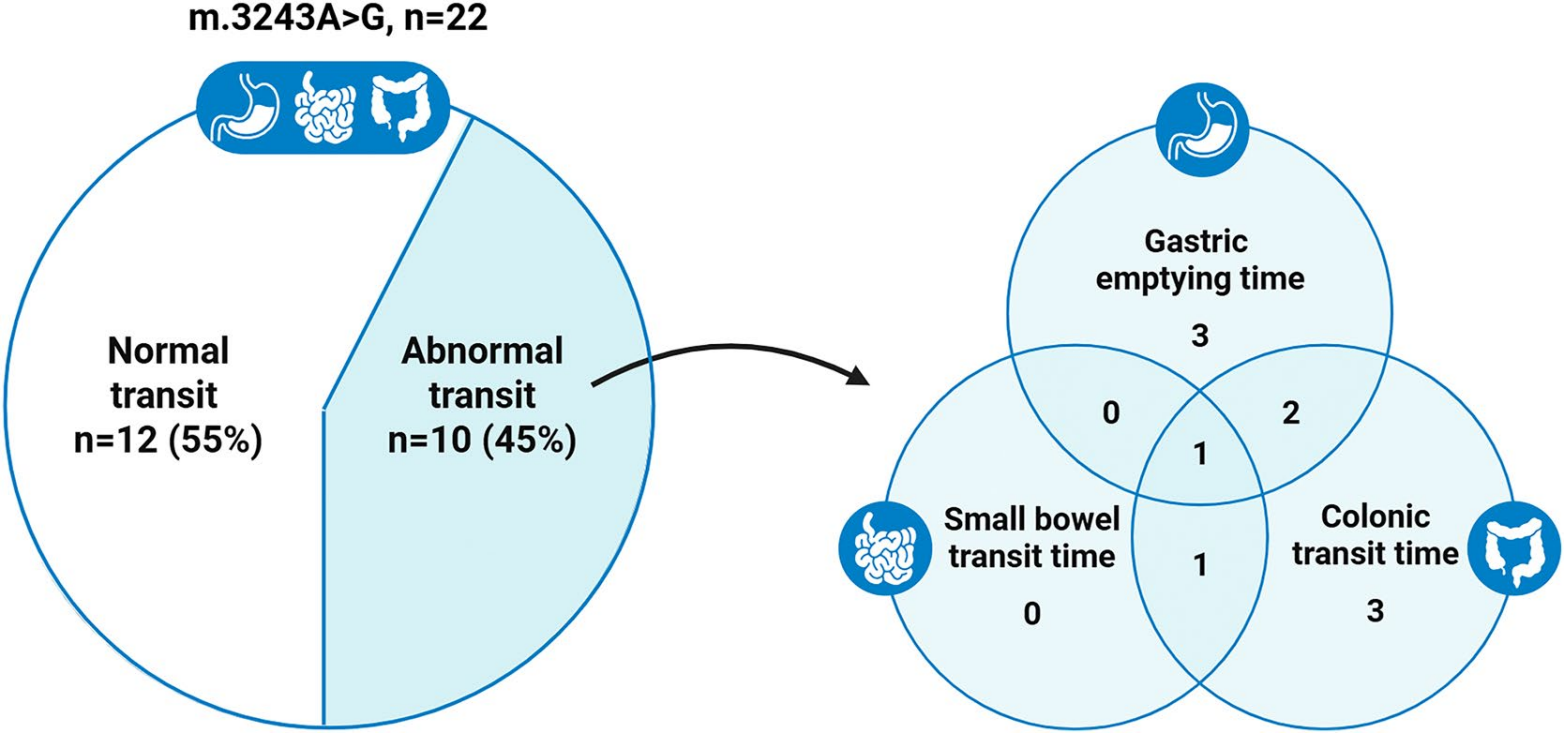
Venn diagram illustrating the distribution of abnormal transit in 10/22 (45%) of m.3243A>G carriers, and if they had single, multi or pansegmental prolonged transit times compared to published reference values.

Compared with healthy controls, m.3243A>G carriers had a significantly prolonged gastric emptying time (221 min [150–348] vs. 165 min [137–199], *p* = 0.02), colonic transit time (2283 min [1082–5153] vs. 1014 min [840–2451], *p* = 0.03) and whole gut transit time (2790 min [1525–5551] vs. 1445 min [1347–2956], *p* = 0.01) (Figure 1, Table 2), which is a prolongation of the median transit time of 34%, 125%, and 93%, respectively. Forty‐five percent of m.3243A>G carriers had a single, multi‐, or pan‐segmental delay in transit time (Figure 2) compared with published gender‐specific reference values [38].

**TABLE 2:** Segmental gastrointestinal transit times, motility indices, pH and questionnaires comparing upper and lower gastrointestinal symptoms in carriers of m.3243A>G and healthy controls.

Category	Variables	m.3243A>G	Controls	*p*
	Number, *n*	22	22	—
Upper GI symptoms assessed by GCSI	Nausea	0.2 (0.0–0.7)	0.0 (0.0–0.0)	**< 0.001**
	Postprandial fullness	1.3 (0.8–2.3)	0.0 (0–0.5)	**< 0.001**
	Bloating	2.3 (0.5–4.0)	0.5 (0.0–1.0)	**0.004**
	Total GCSI‐score	1.3 (0.4–1.9)	0.2 (0.0–0.4)	**< 0.001**
Lower GI symptoms assessed by GSRS	Reflux	1.0 (1.0–3.0)	1.0 (1.0–1.0)	**< 0.001**
	Abdominal pain	1.7 (1.3–3.0)	1.0 (1.0–1.0)	**< 0.001**
	Indigestion	2.8 (1.8–4.0)	1.4 (1.0–1.8)	**< 0.001**
	Diarrhea	2.0 (1.0–4.0)	1.0 (1.0–1.0)	**0.001**
	Constipation	2.0 (1.0–3.0)	1.0 (1.0–1.3)	**< 0.001**
	Total GSRS‐score	2.1 (1.4–3.3)	1.1 (1.0–1.2)	**< 0.001**
Segmental transit times	Gastric emptying time (min)	221 (150–348)	165 (137–199)	**0.02**
	Small bowel transit time (min)	260 (233–339)	310 (241–345)	0.38
	Colonic transit time (min)	2283 (1082–5153)	1014 (840–2451)	**0.03**
	Whole gut transit time (min)	2790 (1525–5551)	1445 (1347–2956)	**0.02**
Motility indices	Gastric (mmHg*s/min)	51.5 (31.7–79.5)	77.6 (45.9–94.3)	0.13
	Small bowel (mmHg*s/min)	118.0 [85.7; 150.2]	152.1 [123.8; 180.4]	0.11
	Colonic (mmHg*s/min)	114.7 [86.2; 143.3]	168.6 [129.9; 207.3]	**0.03**
pH	Gastric pH	1.9 [1.4; 2.4]	2.1 [1.7; 2.6]	0.51
	Trans‐pyloric pH rise	6.1 [5.7; 6.4]	6.2 [6.1; 6.4]	0.41
	Small bowel pH	7.2 [7.0; 7.3]	7.1 [6.9; 7.3]	0.72
	Ileocaecal pH fall	2.1 [1.9; 2.3]	2.1 [1.9; 2.3]	0.61
	Colonic pH	6.6 [6.4; 6.9]	6.8 [6.5; 7.1]	0.37

*Note:* Data are presented as mean [95% confidence interval], median (interquartile range) or number (%). Significant differences are shown in bold.

Abbreviations: GCSI, Gastroparesis Cardinal Symptom Index; GI, gastrointestinal; GSRS, astrointestinal rating score.

### 3.3 Gastrointestinal Motility Index and pH Measurements

The colonic motility index was decreased compared to healthy controls (114.7 mmHg*s/min [86.2; 143.3] vs. 168.6 mmHg*s/min [129.9; 207.3], *p* = 0.03). We did not find any differences in segmental or transpyloric pH rise or ileocaecal pH fall between carriers of m.3243A>G and healthy controls. Data are presented in Table 2.

### 3.4 Gastrointestinal Contractility Pattern

We did find a difference in pressure peak number per hour 30 min before the ileocecal junction where carriers of m.3243A>G mitochondrial disease had a lower number of peaks between 10 and 20 mmHg and above 50 mmHg (*p* = 0.03 and *p* = 0.02, respectively), and above 50 mmHg in the colon (*p* = 0.02). For the largest contractions (peak pressures > 50 mmHg), all segments and transmission sites had a tendency towards lower contractility in the m.3243A>G carrier group (Table 3).

**TABLE 3:** Gastrointestinal contraction pattern analysis in carriers of m.3243A>G and healthy controls along the GI tract.

Intestinal segment or segment transmission site	Contraction pattern (mmHg)	Pressure peak number per hour (number/h)		
		m.3243A>G	Controls	*p*
Stomach	10 < *p* < 20	55.4 [43.2; 67.5]	71.2 [57.2; 85.2]	0.08
Stomach	20 < *p* < 50	12.8 (7.9–23.0)	17.5 (12.6–23.7)	0.45
Stomach	*p* > 50	2.7 (1.3–4.5)	4.3 (2.4–5.5)	0.16
Gastric emptying (30 min before)	10 < *p* < 20	50.6 [30.7; 70.6]	72.6 [43.3; 101.8)	0.20
Gastric emptying (30 min before)	20 < *p* < 50	15.6 [9.7; 21.5]	14.7 [10.1; 19.2]	0.79
Gastric emptying (30 min before)	*p* > 50	6.8 [2.5; 11.1]	10.2 [6.4; 14.0]	0.23
Small bowel	10 < *p* < 20	101.9 [82.4; 121.4]	111.3 [94.1; 128.4]	0.46
Small bowel	20 < *p* < 50	48.4 [30.9; 65.9]	61.0 [45.8; 76.1]	0.27
Small bowel	*p* > 50	2.0 [0.9; 3.0]	2.2 [1.5; 2.9]	0.75
Ileocecal junction (30 min before)	10 < *p* < 20	85.7 [68.1; 103.3]	112.6 [94.6; 130.7]	**0.03**
Ileocecal junction (30 min before)	20 < *p* < 50	89 (20–116)	120 (90–148)	0.15
Ileocecal junction (30 min before)	*p* > 50	4.9 [2.2; 7.6]	10.5 [6.3; 14.7]	**0.02**
Colon	10 < *p* < 20	53.3 [44.1; 62.5]	52.1 [42.6; 61.5]	0.85
Colon	20 < *p* < 50	22.3 [16.0, 28.7]	31.1 [21.8, 40.5]	0.11
Colon	*p* > 50	1.2 (0.8–3.3)	3.6 (2.4–8.0)	**0.02**
Body exit (30 min before)	10 < *p* < 20	80.8 [58.2; 103.3]	72.0 [56.3; 87.7]	0.51
Body exit (30 min before)	20 < *p* < 50	47.3 [32.0; 62.7]	38.5 [24.6; 52.3]	0.38
Body exit (30 min before)	*p* > 50	9.5 [4.3; 14.7]	10.4 [4.1; 16.6]	0.83

*Note:* Contractility data are the number of segmental peaks per hour, being the ratio between the number of peak pressures within the given range and transit time in the gut or transmission region. Gastric emptying, ileocecal junction, and body exit are data obtained 30 min before segment transmission. Data are presented as mean [confidence interval] or median (interquartile range). Significant differences are shown in bold.

Abbreviation: *p*, pressure.

### 3.5 Gastrointestinal Symptoms

The overall questionnaire scores (total Gastroparesis Cardinal Symptom Index‐score and total gastrointestinal rating score) and all sub‐scores (abdominal pain, bloating, constipation, diarrhea, indigestion, nausea and postprandial fullness) besides reflux had a higher median value in the m.3243A>G carrier group compared to healthy controls (Table 2).

### 3.6 Correlation Analysis With Transit Times and Gastrointestinal Parameters, Heteroplasmy Levels and BMI

Whole gut transit time correlated negatively with BMI (*r* = −0.466, *p* = 0.03). We found no correlation between gastric emptying time, colonic transit time, or whole gut transit time and the symptom scores derived from the questionnaires, nor the heteroplasmy levels. Data are presented in Table 4.

**TABLE 4:** Correlation between transit times and symptom scores, gastric motility index, colonic motility index, segmental and segment transmission contraction pattern *p* > 50 mmHg, heteroplasmy levels, and body mass index in carriers of m.3243A>G (*n* = 22), and between transit times and diabetes duration and HbA1c in diabetic m.3243A>G carriers (*n* = 11).

	Gastric emptying time (min)		Colonic transit time (min)		Whole gut transit time (min)	
	*r*	*p*	*r*	*p*	*r*	*p*
Correlations in m.3243A>G carriers (*n* = 22)						
Total GCSI‐score	0.259	0.24	0.186	0.41	0.194	0.39
Total GSRS‐score	0.110	0.62	0.037	0.87	0.027	0.91
Heteroplasmy in blood (%)	0.148	0.51	0.335	0.13	0.348	0.11
Body mass index (kg/m^2^)	−0.204	0.36	−0.402	0.07	−0.466	**0.03**
Correlations in diabetic m.3243A>G carriers (*n* = 11)						
Diabetes duration (years)	0.239	0.48	−0.257	0.44	−0.312	0.35
HbA1c (mmol/mol)	0.193	0.56	−0.216	0.52	−0.216	0.52

Abbreviations: GCSI, Gastroparesis Cardinal Symptom Index; GSRS, gastrointestinal rating score; HbA1c, hemoglobin A1C; *r*, Spearman correlation coefficient. Significant differences are shown in bold.

### 3.7 Influence of Diabetes on the Results

Five out of six (83%) m.3243A>G carriers with prolonged gastric emptying time and six out of seven (86%) with prolonged colonic transit time had diabetes. When comparing the m.3243A>G carriers with and without diabetes to their matched controls, we only found a difference in gastric emptying time, colonic transit time, whole gut transit time, and colonic motility index between m.3243A>G carriers with diabetes compared to their matched controls, and not in the m.3243A>G carriers without diabetes (Table 5). We found no correlations between gastric emptying time, colonic transit time, and whole gut transit time with diabetes duration or HbA1c in diabetic carriers of m.3243A>G (Table 4). The mean blood heteroplasmy level of diabetic m.3243A>G carriers was comparable to the level in nondiabetics (26.8% ± 14.3 vs. 22.3% ± 14.6, *p* = 0.48). The mean age of diabetic carriers was 46 ± 11 years compared to 36 ± 13 years in the nondiabetic group. Other biochemical and demographic differences were a higher HbA1c and fasting glucose in diabetic carriers and a lower BMI and body weight, and all the habitual laxative users have diabetes. In the symptomatic appearance, m.3243A>G carriers with diabetes have a higher frequency of all the addressed symptoms, except for established and borderline cardiovascular autonomic neuropathy, which had the same frequency in diabetic and nondiabetic carriers (see Table S2 for further details).

**TABLE 5:** Differentiation of the study cohort into diabetic vs. nondiabetic m.3243A>G carriers and their age and sex‐matched controls when evaluating differences in segmental transit times and motility indices.

Category	Variables	Diabetic m.3243A>G carriers	Controls	*p*	Nondiabetic m.3243A>G carriers	Controls	*p*
	Number, *n*	11	11	—	11	11	
Segmental transit times	Gastric emptying time (min)	275 (184–1013)	146 (127–171)	**0.005**	177 (146–228)	176 (137–236)	0.87
	Colonic transit time (min)	4182 (2148–6582)	966 (499–1829)	**0.001**	1162 (1007–2330)	1235 (973–2474)	0.74
	Whole gut transit time (min)	4746 (2825–8817)	1379 (934–2227)	**0.002**	1620 (1474–2755)	1602 (1439–2980)	0.92
Motility indices	Colonic (mmHg*s/min)	101.1 [65.4; 136.7]	179.8 [113.2; 246.4]	**0.04**	127.1 [78.3; 176.0]	157.4 [105.9; 208.9]	0.35

*Note:* Data are presented as median (interquartile range) for segmental transit times and mean [95% confidence interval] for motility indices. Significant differences are shown in bold.

## 4 Discussion

The present study demonstrates that in carriers of m.3243A>G, the perceived gastrointestinal symptom burden is high, and dysmotility is evident as increased gastric emptying time, colonic transit time, and whole gut transit time, especially in those patients with concomitant diabetes. There was a trend towards lower segmental motility indices, especially in the colon. No association between these and symptoms could be shown. Data do not allow us to distinguish between myogenic or neurogenic pathoetiology in symptom generation plausibly caused by impaired oxidative phosphorylation, which may be further exaggerated by lifestyle factors.

### 4.1 Gastrointestinal Dysmotility and Nutritional State

Monogenetic mitochondrial disorders are commonly multisystem diseases characterized by significant variability in target organ involvement and phenotypic expression. When evaluating GI symptoms by questionnaires, we could not correlate prolonged segmental transit times to any GI symptoms, including constipation. Gastrointestinal symptoms have multifactorial etiologies, and low levels of physical activity and dietary habits, including inadequate nutritional intake, may also contribute to GI complaints [39, 40]. In addition, pharmacological treatment, that is, antidepressants, antipsychotics, nonsteroidal anti‐inflammatory drugs, and laxatives, is known to affect gut motility [41]. However, for ethical reasons, only laxatives were paused during the study. Furthermore, questionnaires on GI symptoms were obtained 1–2 weeks before SmartPill ingestion, and a recall bias may have been induced. Dysmotility could also be present in mitochondrial patients with only mild GI symptoms, as reported in subjects with the rare autosomal‐recessive mitochondrial neurogastrointestinal encephalomyopathy. Here, subjects with only mild GI symptoms had severe upper GI dysmotility [12]. Dysmotility may result in loss of appetite or episodic dysphagia affecting the overall nutritional state. This is supported by the shown negative correlation between whole gut transit time and BMI, emphasizing that patients with mitochondrial disease often suffer from malnutrition and are underweight [2]. One plausible mechanism is the “ileal brake” a physiological response that activates a feedback mechanism if undigested nutrients reach the ileum, by slowing transit times in the proximal sections of the gut to increase time for nutrients to be available for absorption [42]. In the context of our study, this compensatory mechanism in response to dysmotility may further increase gut stasis, potentially contributing to reduced food intake and, consequently, malnutrition. Additionally, the association of whole gut transit time and BMI indicates constipation, supporting previous findings in m.3243A>G carriers [2]. This is further supported by increased colonic transit times and decreased colonic motility index in our group of m.3243A>G carriers.

### 4.2 Potential Pathological Mechanism of Gastrointestinal Dysmotility

A dual pathoethiology of GI dysmotility in m.3243A>G could be either of neurogenic and/or myogenic origin. Notably, we did not find a correlation between mitochondrial heteroplasmy levels in blood and segmental transit times. This lack of correlation may be caused by a relatively wide range in heteroplasmy levels as a proxy for disease severity, thus causing a type II error. It may, however, also indicate that other mechanisms than impaired mitochondrial ATP production are associated with GI neuromuscular dysfunction in carriers of m.3243A>G.

Carriers of the m.3243A>G variant have a high risk of developing diabetes [43], a well‐known risk factor for autonomic neuropathy leading to gastroparesis and GI dysmotility [44]. Thus, although there was an increase in gastric emptying time, colonic transit time, and whole gut transit time and a decrease in colonic motility index in m.3243A>G carriers with diabetes, there were no differences between m.3243A>G carriers without diabetes and sex‐matched controls. This suggests that concomitant diabetes, plausibly entailing diabetic autonomic neuropathy, synergistically impacts the neuromuscular GI function, leading to more severe GI dysmotility. In type 1 and 2 diabetes, hyperglycemia causes pan‐intestinal dysmotility due to structural remodeling of the gut wall and impairment of the enteric nerves regulating the GI milieu [44, 45, 46, 47]. Enteric nerves are especially vulnerable to hyperglycemia, which damages the neurons. In addition, various hyperglycemia‐associated metabolic pathways induce neurotoxicity, neuroinflammation, and oxidative stress, all with adverse effects on neurons [44]. However, the presence of diabetes is also associated with an overall more severe symptomatic presentation of m.3243A>G disease [43]. This is also reflected in our dataset, where diabetic m.3243A>G carriers perceive a higher symptom burden than nondiabetic carriers. There were no associations between diabetes duration or HbA1c and any of the prolonged transit times, and diabetic autonomic neuropathy can already be present at the time of diagnosis of type 2 diabetes [48], whereas HbA1C only provides a time‐limited measure of overall glucose control. Thus, the pathophysiology leading to differences in segmental transit times is only partly explained by hyperglycemic conditions, as diabetic patients could represent subjects more severely affected by their mitochondrial disease. This is supported by m.3243A>G carriers having a higher prevalence of the microvascular complication established cardiac autonomic neuropathy, of which only half had diabetes, indicating that the mitochondrial dysfunction leads to autonomic neuropathy. In addition to the proposed neurogenic dysfunction, decreased smooth muscle function in the gut wall could contribute to dysmotility. Especially given the observed trend in the contractile pattern, where the frequency of the largest gut muscle contractions (> 50 mmHg) is reduced across all gut segments and transit phases, with a pronounced reduction in the colon and prior to the ileocecal junction.

### 4.3 Study Limitations

This study has several limitations. First, the study cohort is small, which may have resulted in type II errors due to insufficient statistical power. Additionally, the exploratory nature of the study, requiring multiple statistical tests, raises the risk of mass significance. Furthermore, no power calculations were performed. Second, we may have induced selection bias as we were not able to include m.3243A>G carriers suffering from severe dysphagia or the severe phenotype mitochondrial encephalomyopathy with lactate acidosis and stroke‐like episodes. This could exclude the m.3243A>G carriers potentially suffering from the most burdensome GI symptoms. Third, the study included a high percentage of females. Females are generally known to have longer GI transit times than males [38], which may overestimate the presence of delay in transit times.

## 5 Conclusion

In conclusion, we have provided novel insight into GI dysmotility in m.3243A>G carriers using the SmartPill. Increasing evidence indicates that GI dysmotility in m.3243A>G carriers may result from a combination of neurogenic and myogenic impairment exaggerated by lifestyle factors. Our findings indicate that the diabetic carriers are most severely affected by GI dysmotility; however, it is unknown whether this is caused by a direct effect of their mitochondrial disease or induced by diabetic autonomic neuropathy. Future treatment options for impaired oxidative phosphorylation may prevent damage to autonomic nerves or increase intestinal smooth muscle function in carriers of m.3243A>G with a potential positive effect on patients suffering from GI disturbances.

## Author Contributions

S.R.N., A.L.F., and C.B. conceived the study. S.R.N. performed the study, and S.R.N. and C.B. analyzed the SmartPill data. M.P.S. measured heteroplasmy levels. D.L. performed contractility pattern analysis. S.R.N. analyzed the data. S.R.N., A.L.F., and C.B. wrote and revised the manuscript. All authors contributed to data interpretation and approved the final version of the manuscript.

## Conflicts of Interest

The authors declare no conflicts of interest.

## Supporting information


Appendix S1.

## Data Availability

The data that support the findings of this study are available on request from the corresponding author. The data are not publicly available due to privacy or ethical restrictions.
