# Supplementary material for: Prolonged Gastrointestinal Transit Times and Dysmotility in m.3243A>G Mitochondrial Disease
Source: Neurogastroenterol Motil. 2025 Jun 4;37(12):e70092. doi: 10.1111/nmo.70092 (PMC12623286; doi:10.1111/nmo.70092)
Supplement: Supplementary file 1 — Appendix S1. [file NMO-37-e70092-s001.docx]

**Prolonged Gastrointestinal Transit Times and Dysmotility in m.3243A>G Mitochondrial Disease**

**Supporting information**

**1) Heteroplasmy level in blood**

*DNA isolation*

DNA for heteroplasmy analysis was isolated from blood for all 22 m.3243A>G carriers. DNA from blood was isolated using QIAsymphony DSP DNA midi Kit (Qiagen, Hilden, Germany) according to the manufacturer’s instructions.

*Heteroplasmy level in blood*

The QX200™ Droplet Digital PCR (polymerase chain reaction) system (Bio-Rad, Hercules, California, USA) was used to quantify the fractional abundance of mitochondrial DNA with m.3243A>G. The 22 µl Droplet Digital PCR mixture consisted of 11 µl 2x Droplet Digital PCR SuperMix for probes (no dUTP) (Bio-Rad, Hercules, California, USA), 909 nM of each primer, 284 nM of each probe and 5 µl template DNA (forward primer: CCGTAAATGATATCATCTCA, reverse primer: CTCTGACTGTAAAGTTTTAAG, wild type probe: Fam-ACCGGGCTCTGCCAT-3'-BHQ-1, mutant probe: Hex-ACCGGGCCCTGCCAT-3'-BHQ-1). Droplets were generated using an Automated Droplet Generator (Bio-Rad, Hercules, California), transferred to a semi-skirted 96-well PCR plate and sealed with pierceable foil (Bio-Rad, Hercules, California). The PCR was performed on a CFX96 Touch Deep Well Real-Time PCR System (Bio-Rad, Hercules, California) with the following conditions: 95°C for 10 minutes, 50 cycles of 94°C for 30 seconds and 56°C for 1 minute, and 98°C for 10 minutes with a ramp rate of 1°C/s. The plate was kept on hold at 4°C for a minimum of 30 minutes or at 12°C for a minimum of 4 hours as described previously (Rowlands et al. 2019) followed by 10 minutes of incubation at room temperature before the fluorescence signals were measured by the QX200™ Droplet Reader (Bio-Rad, Hercules, California). Each experiment included a positive template control run in duplicates and a negative template control run in five wells as well as three no-template controls (NTC).

For data analysis, only wells with a minimum of 10,000 accepted droplets were analyzed. Thresholds were manually set based on positive and negative controls with gating based on fluorescence amplitude in 1D and 2D plots. Data was analyzed with QX Manager Software 2.0 (Bio-Rad, Hercules, California). The fractional abundance was calculated based on the concentration of both m.3243A>G and wild-type mitochondrial DNA for each sample.

**2) Table S1** List of drugs taken by m.3243A>G carriers and healthy controls

| **Drug** | **m.3243A>G** | **Controls** |
| --- | --- | --- |
| Number, *n* | 22 | 22 |
| Angiotensin converting enzyme inhibitors/Angiotensin receptor blockers (yes), *n* | 7 (32%) | 1 (5%) |
| Antihistamines (yes), *n* | 2 (9%) | 1 (5%) |
| Antipsychotics (yes), *n* | 3 (14%) | 0 (0%) |
| Antiviral medication (yes), *n* | 1 (5%) | 0 (0%) |
| Arginine supplement (yes), *n* | 1 (5%) | 0 (0%) |
| Beta-blockers (yes), *n* | 2 (9%) | 0 (0%) |
| Bisphosphonates (yes), *n* | 3 (14%) | 1 (5%) |
| Bronchodilators (yes), *n* | 3 (14%) | 0 (0%) |
| Calcium channel blockers (yes), *n* | 2 (9%) | 1 (5%) |
| Calcium supplement (yes), *n* | 5 (23%) | 1 (5%) |
| Coenzyme Q_10_ (yes), *n* | 1 (5%) | 0 (0%) |
| Denosumab (yes), *n* | 1 (5%) | 0 (0%) |
| Dipeptidyl peptidase-4 inhibitors (yes), *n* | 3 (14%) | 0 (0%) |
| Diuretics (yes), *n* | 1 (5%) | 0 (0%) |
| Ezetimibe (yes), *n* | 1 (5%) | 0 (0%) |
| Fish oil supplement (yes), *n* | 2 (9%) | 2 (9%) |
| Gabapentin (yes), *n* | 1 (5%) | 0 (0%) |
| Insulin (yes), *n* | 6 (27%) | 0 (0%) |
| Laxatives† (yes), *n* | 5 (23%) | 0 (0%) |
| Lamotrigine (yes), *n* | 1 (5%) | 0 (0%) |
| Leukotriene receptor antagonists (yes), *n* | 2 (9%) | 0 (0%) |
| Lithium citrate (yes), *n* | 1 (5%) | 0 (0%) |
| Magnesium supplement (yes), *n* | 1 (5%) | 1 (5%) |
| Melatonin (yes), *n* | 1 (5%) | 0 (0%) |
| Metformin (yes), *n* | 1 (5%) | 0 (0%) |
| Methotrexate (yes), *n* | 1 (5%) | 0 (0%) |
| Multivitamin supplement (yes), *n* | 4 (18%) | 3 (14%) |
| Nasal corticosteroids (yes), *n* | 4 (18%) | 1 (5%) |
| Nonsteroidal anti-inflammatories (yes), *n* | 3 (14%) | 0 (0%) |
| Paracetamol (yes), *n* | 4 (18%) | 0 (0%) |
| Potassium supplement (yes), *n* | 1 (5%) | 0 (0%) |
| Proton-pump inhibitors (yes), *n* | 2 (9%) | 0 (0%) |
| Selective serotonin reuptake inhibitors (yes), *n* | 1 (5%) | 0 (0%) |
| Serotonin and norepinephrine reuptake inhibitors (yes), *n* | 1 (5%) | 0 (0%) |
| Skeletal muscle relaxants (yes), *n* | 1 (5%) | 0 (0%) |
| Statins (yes), *n* | 6 (27%) | 1 (5%) |
| Tricyclic antidepressants (yes), *n* | 1 (5%) | 0 (0%) |
| Vitamin B supplement (yes), *n* | 2 (9%) | 0 (0%) |
| Vitamin D supplement (yes), *n* | 6 (27%) | 3 (14%) |

† Laxatives were paused three days prior to the investigation.

**3) Table S2:** Demographic characteristics of carriers of m.3243A>G with and without diabetes. Data are presented as mean ± standard deviation or median (interquartile range) or number (%). Significant differences are marked with *. Normal reference values for biochemistry results are noted in brackets.

| **Category** | **Variables** | **Diabetic m.3243A>G carriers** | **Non-diabetic m.3243A>G carriers** | **p-value** |
| --- | --- | --- | --- | --- |
|  | Number, *n* | 11 | 11 | - |
| *Basic characteristics* | Sex (Female/male), *n* | 9/2 | 6/5 |  |
|  | Age (years) | 46 ±11 | 36 ± 13 | 0.08 |
|  | Current smokers (yes), *n* | 4 | 2 |  |
| *Physical examination* | Weight (kg) | 57.1 ± 10.2 | 72.1 ± 19.1 | **0.03*** |
|  | Height (cm) | 166 ± 7 | 171 ± 10 | 0.24* |
|  | Body mass index (kg/m^2^) | 20.1 (17.8-23.8) | 23.5 (21.9-27.0) | **0.04*** |
|  | Established CAN (yes), *n* | 5 (45%) | 5 (45%) | 1.00 |
|  | Borderline CAN (yes), *n* | 3 (27%) | 3 (27%) |  |
|  | No CAN (yes), *n* | 3 (27%) | 3 (27%) |  |
| *Biochemistry* | Heteroplasmy in blood (%) | 26.8 ± 14.3 | 22.3 ± 14.6 | 0.48 |
|  | p-hemoglobin [men 8.3-10.5, women 7.3-9.5 mmol/L] | 8.5 ± 1.0 | 9.0 ± 1.2 | 0.28* |
|  | p-creatinine [men 60-105, women 45-90 µmol/L] | 71 ± 17 | 67 ± 11 | 0.58 |
|  | p-lactate [0.5-2.5 mmol/l] | 1.6 (1.2-2.1) | 1.3 (1.2-1.9) | 0.36 |
|  | HbA1c [31-44 mmol/mol] | 45 (44-60) | 36 (32-42) | **<0.001*** |
|  | p-glucose [4.2-7.8 mmol/L] | 7.1 (6.9-9.1) | 6.2 (5.5-7.2) | **0.009*** |
|  | p-cholesterol [<5.0 mmol/L] | 4.3 ± 0.9 | 4.6 ± 0.9 | 0.37 |
|  | p-triglycerides [<2.0 mmol/L] | 1.2 (1.0-1.6) | 1.5 (1.1-1.8) | 0.22 |
| *Medication* | Habitual use of laxatives (yes), *n* † | 5 (45%) | 0 (0%) | - |
| *Symptomatic assessment* | Cardiomyopathy (yes)*, n* | 4 (36%) | 0 (0%) | - |
|  | Hypertension (yes)*, n* | 5 (45%) | 2 (18%) | - |
|  | Hearing impairment (yes)*, n* | 10 (91%) | 4 (36%) | - |
|  | Myopathy (yes)*, n* | 9 (82%) | 5 (45%) | - |
|  | Ataxia (yes)*, n* | 2 (18%) | 0 (0%) | - |
|  | Epilepsy (yes)*, n* | 0 (0%) | 0 (0%) | - |
|  | Stroke-like episodes (yes)*, n* | 0 (0%) | 0 (0%) | - |
|  | Non-diabetic nephropathy (yes)*, n* | 1 (9%) | 0 (0%) | - |
|  | Peripheral neuropathy (yes)*, n* | 5 (45%) | 3 (27%) | - |

Abbreviations: CAN – cardiovascular autonomic neuropathy; p – plasma; HbA1c – hemoglobin A1C

†Laxatives were paused three days prior to the investigation.
